# Can Radiologists Replace Digital 2D Mammography with Synthetic 2D Mammography in Breast Cancer Screening and Diagnosis, or Are Both Still Needed?

**DOI:** 10.3390/diagnostics14212452

**Published:** 2024-11-01

**Authors:** Areej Saud Aloufi, Mona Alomrani, Rafat Mohtasib, Bayan Altassan, Afaf Bin Rakhis, Mehreen Anees Malik

**Affiliations:** 1 Radiological Sciences Department, College of Applied Medical Sciences, King Saud University, Riyadh 12371, Saudi Arabia; 2 Women’s Imaging, Radiology Department, King Khaled University Hospital, King Saud University, Riyadh 12372, Saudi Arabiabaltassan@ksu.edu.sa (B.A.); mehreen4110@gmail.com (M.A.M.); 3 College of Medicine, Alfaisal University, Riyadh 11533, Saudi Arabia; rmohtasib@alfaisal.edu

**Keywords:** breast cancer, digital mammography, synthetic mammography, cancer visibility, breast density and cancer size

## Abstract

**Background/Objectives**: Digital mammography (DM) has long been the standard for breast cancer screening, while digital breast tomosynthesis (DBT) offers an advanced 3D imaging modality capable of generating 2D Synthetic Mammography (SM) images. Despite SM’s potential to reduce radiation exposure, many clinics favor DM, with DBT and SM, due to its perceived diagnostic reliability. This study investigates whether radiologists can replace DM with SM in breast cancer screening and diagnosis or if both modalities are necessary. **Methods**: We retrospectively analyzed DM and SM images from 375 women aged 40–65 who underwent DM with DBT at King Khaled University Hospital from 2020–2022. Three radiologists evaluated the images using ACR BI-RADS, assessing diagnostic accuracy via the area under the receiver operating characteristic (ROC) curve (AUC). The agreement in cancer conspicuity, breast density, size, and calcifications were measured using weighted kappa (κ). **Results**: Among 57 confirmed cancer cases and 290 cancer-free cases, DM demonstrated higher sensitivity (82.5% vs. 78.9%) and diagnostic accuracy (AUC 0.800 vs. 0.783, *p* < 0.05) compared to SM. However, SM detected more suspicious calcifications in cancer cases (75.6% vs. 51.2%, *p* < 0.05). Agreement was fair for conspicuity (κ = 0.288) and calcifications (κ = 0.409), moderate for density (κ = 0.591), and poor for size (κ = 0.254). **Conclusions**: while SM demonstrates enhanced effectiveness in detecting microcalcifications, DM still proves superior in overall diagnostic accuracy and image clarity. Therefore, although SM offers certain advantages, it remains slightly inferior to DM and cannot yet replace DM in breast cancer screening.

## 1. Introduction

Breast cancer is one of the leading causes of cancer-related deaths among women worldwide and in Saudi Arabia [1,2,3]. Early detection through breast imaging plays a crucial role in improving patient outcomes. In recent years, digital breast tomosynthesis has been introduced as an advanced imaging technique for breast cancer screening. DBT, approved by the FDA in 2011, provides a three-dimensional view of the breast, allowing for better visualization and improved detection of lesions [4]. Traditionally, digital mammography DM has been the standard imaging modality for breast cancer screening. However, with the introduction of DBT, there has been a shift towards using synthesized 2D images generated from DBT scans as an alternative to reduce radiation dose and time while maintaining diagnostic accuracy [5].

Despite the benefits of DBT and synthetic 2D mammography (SM), many breast imaging and screening clinics still prefer digital mammography (DM) images over synthetic 2D images in diagnosing breast lesions [6]. This preference is primarily due to radiologists feeling more confident interpreting DM images—radiologists reported that 2D views of DM are much sharper with fewer artefacts—and in addition, there may be concerns about the perceived diagnostic accuracy of synthetic 2D images compared to DM in screening [6]. Moreover, this preference for DM images over SM 2D images is not limited to a few clinics but is observed across several breast imaging and screening centers in Saudi Arabia.

However, performing both DM and DBT for each screening or diagnostic exam increases radiation dose to patients and diagnosis time [4]. The comparison between 2D DM and 2D SM in breast cancer diagnosis and detection is a topic of significant interest in the field of breast imaging. Digital mammography and digital breast tomosynthesis (DBT) are widely used for breast cancer screening, with studies demonstrating a reduction in breast cancer-related mortality of 20–50% [7]. Digital mammography has been reported to have a high sensitivity of 97% and a specificity of 64.5% in breast cancer detection [8]. However, it is also known to miss 10–15% of breast cancers, which can be attributed to intrinsic growth patterns of select cancers, technical factors, and the failure of radiologists to detect very subtle signs of malignancy [6].

Two-dimensional SM without DBT has been compared to mammography in breast cancer diagnosis. Studies have shown that 2D SM combined with DBT (SM/DBT) has similar diagnostic performance to DM combined with DBT (DM/DBT) in terms of breast cancer detection rate (CDR) and specificity [9,10]. However, SM/DBT has been found to have a lower recall rate and biopsy rate compared to DM/DBT, indicating a potential reduction in unnecessary recalls and biopsies [11]. In addition, SM/DBT has been shown to improve CDR and reduce recall rate compared to mammography alone [12]. The use of SM/DBT in breast cancer screening may therefore be a viable alternative to DM/DBT, offering similar diagnostic performance with reduced radiation dose [13].

Zuley et al. (2014) conducted a retrospective observer performance study to assess the interpretation performance and radiation dose of 2D SM images versus standard DM images alone or in combination with digital breast tomosynthesis images [14]. The study included 123 cases and 8 readers, and the accuracy of probability of malignancy ratings and seven-category forced BI-RADS ratings were evaluated. The results showed that the probability of malignancy-based mean AUCs for SM and DM images alone was 0.894 and 0.889, respectively (difference, 0.005; 95% confidence interval [CI]: −0.062, 0.054; *p* = 0.85). The mean AUC for SM with tomosynthesis and DM with tomosynthesis was 0.916 and 0.939, respectively (difference, 0.023; 95% CI: −0.011, 0.057; *p* = 0.19). The study concluded that SM alone or in combination with tomosynthesis is comparable in performance to DM alone or in combination with tomosynthesis and may eliminate the need for DM as part of a routine clinical study.

Alabousi et al. (2020) conducted a systematic review and meta-analysis comparing the performance of DBT, SM, and DM in breast cancer screening [15]. The study included 42 studies with 2,606,296 patients and found that the cancer detection rate (CDR) was highest in combined DBT and SM, with a CDR of 7.40 per 1000 screened. However, invasive CDR was highest in combined DBT and DM (4.53 per 1000 screened, 95% CI = 3.97 to 5.12, *p* = 0.003) and combined DBT and SM (5.68 per 1000 screened, 95% CI = 4.43 to 7.09, *p* < 0.001). The study’s results suggest that the combination of DBT and SM may offer advantages in terms of cancer detection and recall rates compared to DM alone.

However, to the authors’ knowledge, no study has compared the diagnostic accuracy of DM and SM alone in cancer detection and diagnosis, nor assessed the agreement in cancer visibility, breast density, and cancer size. Therefore, the main aim of this study was to conduct a comparison with a sufficient sample that includes cancer cases and normal cases from Saudi women attending breast screening clinics and to directly compare DM and SM in terms of diagnostic accuracy and cancer detection and agreement level in cancer conspicuity, breast density and cancer size.

## 2. Materials and Methods

Ethical approval was received from the IRB at King Saud University KSU (project No. E-22-7308) to conduct a comparative study that assesses the accuracy of SM compared to DM in breast cancer detection and diagnosis, in addition to evaluating the agreement between the two methods in cancer conspicuity, breast density, and cancer size measurement. The study collected anonymized DM and SM images of women between 40 and 65 years old who underwent DBT with mammography at King Khaled University Hospital KKUH in Riyadh, Saudi Arabia between 2020 and 2022. The study excluded women with a previous history of breast cancer or who underwent unilateral mastectomy or previous breast surgeries.

Right and left Craniocaudal CC and Medio lateral Oblique MLO images of mammography and DBT were acquired in the same position and compression. Thus, mammography and the reconstructed SM were all acquired at the same time. All images were taken by the Fujifilm 3D digital mammography system and assessed on a 4k reading station at KKUH. SM and DM Images from the same women were randomly and blindly assessed by three breast imaging radiologists.

The findings were evaluated by radiologists using the ACR BI-RADS for breast cancer diagnosis 5th edition [16], which was modified into a one-page reporting form for each modality.

Test findings were categorized as negative (BIRADS categories 1, 2, or 3) or positive (BIRADS categories 4 or 5) to analyze the diagnostic yield of SM and DM—this test cutoff was chosen because of the fact that the sample was obtained from a diagnostic clinic and this complies with their clinical practice. A BI-RADS score was assigned for each woman’s exam; in cancer cases, the score of the malignant lesion was considered, while for cancer-free women, the lesion with the highest BI-RADS score or normal results were used. However, for cancer cases, (the index cancer BI-RADS score) and cancer-free women, only one lesion per woman was used in the analysis.

In the one-page reporting form, radiologists were asked to report suspicious lesions and provide a BI-RADS category for each lesion, in addition to evaluating the visibility of cancer, breast density, and cancer size if seen.

To assess the diagnostic accuracy of SM compared to DM, the pathological findings were regarded as reference or gold standard. Women with a proven malignant finding in histopathology (DCIS, IDC, LCIS, ILC, and/or invasive mammary carcinoma) were classified as cancer cases; any additional pathology findings were labeled as non-malignant. Cancer-free women were defined as those who were recalled but found to be cancer-free in histopathology or not recalled but had a normal follow-up exam after at least two years.

Based on the gold standard results, positive and negative data from each imaging modality were classified as true positive (TP), true negative (TN), false positive (FP), or false negative (FN).

Thus, Sensitivity, Specificity, Positive Likelihood Ratio, Negative Likelihood Ratio, Positive Predictive Value, Negative Predictive Value, and Accuracy were calculated for SM and DM, in addition to establishing the accuracy of each modality by comparing with a non-inferiority null hypothesis based on the difference in the area under the receiver operating characteristic (ROC) curve (AUC). To assess the statistical significance of results Pearson’s Chi-square test was used in the differentiation between cancer cases and cancer-free women for SM and DM; a *p*-value < 0.05 was considered significant.

Frequency tables were provided for SM and DM for Cancer Conspicuity, breast density, and cancer size. Cancer Conspicuity was reported as not visible, barely visible, visible but not well seen, or clearly visible. Breast density assessment for each woman was grouped either non-dense (ACR BIRADS A and B) or dense (ACR BIRADS C and D). Results of cancer size were grouped into cancer sizes from 0 to 1 cm, more than 1 cm, up to 2, more than 2 cm, or cancer was not measured by radiologists. To assess the agreement between the two methods, the correlation using weighted Kappa (κ) was used. The strength of the agreement was defined according to the five categories of Landis and Koch (1977): slight 0.00–0.20; fair 0.21–0.40; moderate 0.41–0.60; substantial 0.61–0.80; excellent 0.81–1.00 [17]. All results were generated using SPSS IBM software version 29, and a *p*-value (2-tailed) < 0.05 was considered statistically significant.

## 3. Results

The study sample included 375 women who underwent mammography and digital breast tomosynthesis at KKUH hospital between 2020 and 2022; the sample was found to be 57 pathology-proven cancer cases and 290 women with normal results (normal follow-up mammography exams after two years of benign biopsy results). The remaining 28 women were diagnosed by two consultant radiologists as BI-RADS four or five; however, they did not show up for any additional imaging or biopsy. Table 1 shows the study sample characteristics.

### 3.1. Accuracy of SM Compared to DM in Breast Cancer Detection and Diagnosis

**Figure 1:**
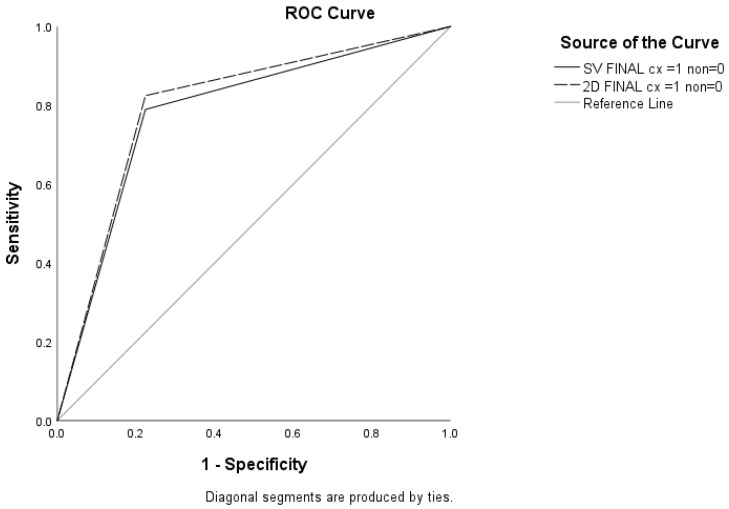
Receiver operating characteristic curves for DM and SM and DBT. The operating points at BI-RADS 4 for all imaging modalities are indicated.

Results here will be presented for 57 cancer cases and 290 benign or normal cases; 28 non-confirmed cancer cases are excluded because they were without reference standard (biopsy or normal two-year follow-up exam). The cancer detection result for DM was found to be higher than SM (47/57 82.5% vs. 45/57 78.9%). Therefore, diagnostic accuracy was higher for DM, with AUC 0.800 (95% CI = 0.737–0.864) compared to 0.783 (95% CI 0.715–0.850), *p* < 0.05 (Figure 1).

Among the 57 cancer cases, 41 were reported in clinical practice after DBT, SM, and DM reading that they have suspicious microcalcifications and were considered the true positive in this analysis. The remaining 16 are cancer cases without reported microcalcification. In this study, SM detected more suspicious microcalcifications than DM [31/41 (75.6%) vs. 21/41 (51.2%), *p* < 0.05]. The agreement between the two methods in microcalcifications detection is reported in the next section of the results.

Diagnostic accuracy results for SM and DM were comparable, and the specificity of each method was similar; however, the sensitivity of DM was higher than that of SM, with 82.46% vs. 78.95%, respectively (*p* < 0.05). Table 2 shows the results of diagnostic accuracy parameters including Sensitivity, Specificity, Positive Likelihood Ratio, Negative Likelihood Ratio, PPV, NPV, and Accuracy.

### 3.2. Agreement Between Mammography and Synthetic Mammography in Cancer Visibility, Breast Density, Cancer Size, and Microcalcification Detection

Among 57 cancer cases, radiologists clearly visualized cancer on 45 from DM images compared to 36 for SM, however, 4 cancer cases were not visible compared to 2, respectively (Table 3), the agreement between the methods in conspicuity was fair k = 0.288 (Table 4).

The sample was grouped into dense and non-dense to assess the agreement between the two methods in breast density assessment. The two methods showed the highest agreement results in breast density, and the result was almost substantial, with k = 0.591; 173 women were considered to have dense breasts compared to 148, and the difference was found to be statistically significant *p* < 0.05.

The results in cancer size were not close between SM and DM, with the lowest agreement result of k = 0.254, but a better agreement was found in microcalcification detection (k = 0.409); however, as reported previously, SM detected more than DM (Table 4).

## 4. Discussion

**Figure 2:**
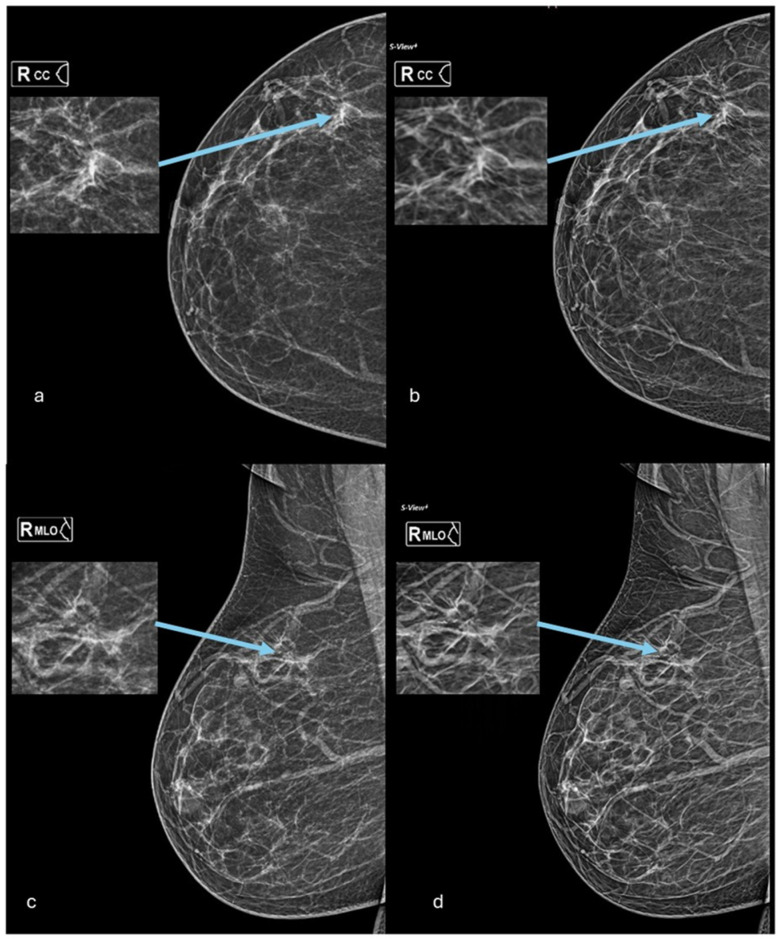
DM and SM images of the right breast for a 62-year-old woman who attended an annual screening at KKUH and was diagnosed with breast cancer. She had no signs or symptoms, only a family history of breast cancer (one sister at the age of 48). In this study, the radiologist who read images from 2D mammography only reported a clearly visible focal distortion (arrows in images (**a**,**c**)) and reported the case to be a BI-RADS 4 (suspicious). However, the SM-only results showed no suspicious areas in the corresponding location (arrows in images (**b**,**d**)), only a barely visible circumscribed mass in the RCC, and it was considered a benign case (BI-RADS 2).

Breast cancer is one of the leading causes of cancer-related deaths among women worldwide. Early detection of breast cancer plays a crucial role in improving survival rates and reducing mortality [18]. Therefore, it is essential to evaluate and compare different imaging modalities to determine their effectiveness in detecting breast cancer. In this study, we compared the diagnostic performance of two imaging techniques—2D digital mammography (DM) and 2D synthetic mammography (SM)—in breast cancer diagnosis, cancer visibility, calcification detection, breast density, and cancer size. Our results showed that in cancer diagnosis and detection, DM was superior to SM. This finding is consistent with previous studies that have demonstrated the superiority of DM in terms of cancer detection and lesion diagnosis. The higher cancer detection rate for DM may be attributed to its ability to provide clearer and more detailed images of breast tissue. Although specificity in cancer diagnosis was similar for both methods, DM showed better sensitivity than SM (82.46% vs. 78.95%, respectively), and thus the diagnostic accuracy, as measured by the area under the curve, was significantly higher for DM. Although in the literature the two methods were found to be comparable, a systematic review by Abdullah et al. (2020) found that SM was superior in accuracy to DM, their results showed that SM has slightly better sensitivity than DM (75% vs. 73%) and much higher specificity (92% vs. 88%) [19]. Additionally, the results suggest that DM is superior in cancer visibility and thus may have an advantage in detecting smaller or less conspicuous cancer lesions (Figure 2). These findings are consistent with previous studies that have shown the superior performance of DM in detecting breast cancers, especially those that are small or less conspicuous [20].

**Figure 3:**
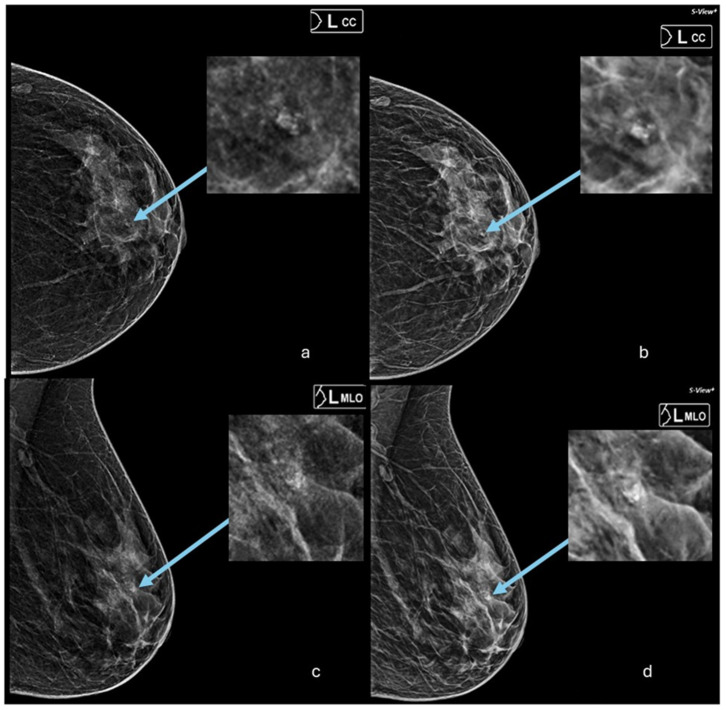
Two-dimensional and SM images of the left breast for a 48-year-old woman who attended an annual screening at KKUH and was diagnosed with breast cancer. In this study, the radiologist who assessed images from SM reported a barely visible microcalcification in LCC (**b**) and visible, not well-seen microcalcification in the LMLO image (**d**). On the other hand, the radiologist who read the 2D-only image in this retrospective analysis did not report microcalcification from LCC (**a**) or LMLO (**c**) of the 2D mammography. The arrows in the images indicate the microcalcification locations.

On the other hand, in terms of microcalcification detection, SM was found to detect more suspicious microcalcifications in cancer cases than DM (Figure 3). Microcalcifications are often an early indicator of ductal carcinoma in situ (DCIS), a precursor to invasive breast cancer. By enhancing the visibility of these calcifications, SM could potentially lead to earlier and more accurate diagnoses, allowing for more timely interventions and improved patient outcomes. Although our results showed that the agreement between the two methods in microcalcification detection was reported to be fair, this finding aligns with previous research, especially one study by Dodelzon et al. (2020) which aimed to compare the performance of SM and DM in the detection of microcalcifications. They included images from 160 patients, and the results showed that there was no difference in diagnostic accuracy between SM and DM, with comparable area under the curve (AUC) values of 91% and 88%, respectively. However, SM had lower specificity and positive predictive value compared to DM. Radiologists found calcifications to be more conspicuous on SM, but there was no significant difference in subjective diagnostic confidence [20]. Based on these findings, SM was determined to be non-inferior to DM in the detection of microcalcifications, with increased conspicuity but comparable diagnostic confidence.

Accurate assessment of tumor size, margins, and treatment response is crucial in breast cancer management, particularly for determining the appropriate surgical approach and ensuring complete resection of malignant tissue [21]. Digital mammography (DM), despite its 2D nature, provides valuable information about tumor presence but often falls short in visualizing the full extent of the disease. It has been found that digital breast tomosynthesis (DBT) and synthetic mammography (SM) are more accurate than DM in measuring tumor sizes, with a higher correlation to histological tumor sizes (ρ = 0.887 and 0.852 for DBT and SM, respectively, compared to 0.814 for DM; *p* < 0.001) [22]. Another study indicated that DBT assessments were more accurate than DM, with a success rate of 69% compared to 40% for DM in identifying correct tumor margins [23].

However, a more recent meta-analysis showed that both DM and DBT have lower accuracy than ultrasound in intraoperative tumor margin assessment [24]. Breast ultrasonography demonstrated a 99.5% assessment rate for tumors post-neoadjuvant chemotherapy, significantly outperforming mammography, which had an assessment rate of 84.5% [25]. Furthermore, while mammography showed superior accuracy in estimating tumor size, it failed to predict pathological complete response (pCR) or tumor-free margins with high confidence [25]. This limitation underscores the importance of a multimodal imaging approach.

As discussed in a review, ultrasound and MRI are considered more reliable methods than radiography techniques for assessing tumor response after neoadjuvant chemotherapy. These modalities not only measure residual tumor size more accurately but also provide essential insights into tumor characteristics, which are critical for guiding treatment decisions [26].

Our results also showed good breast density agreement between SM and DM. Breast density is an important risk factor for breast cancer; women who are considered to have dense breasts are advised to have additional MRI or ultrasound for cancer screening, and therefore agreement between the two methods is important if SM were to be considered for replacing DM in screening. Alshafeiy et al. (2017) compared breast density assessments between SM and DM. They found, after three radiologists assessed the breast density of 309 women using BI-RADS, that the agreement between consensus density categories for the two methods was 80.3%, with a kappa coefficient of 0.73. Overall, SM was found to be comparable to DM in assessing breast density. However, there were some variations in density assessment between individual readers. The study suggests that practices can adopt SM without concerns about its impact on density assessment and subsequent recommendations for supplemental screening [27].

Regarding cancer size, the agreement between SM and DM was low, indicating discrepancies in the assessment of cancer size between the two methods [28]. This emphasizes the need for further research and the development of robust measurement techniques for lesion size, as highlighted in the literature [29,30].

Overall, from the results of this study and previous literature, SM is not yet considered a reliable substitute for DM in cancer screening and diagnosis; further advancement in post-processing could help in improving the images of SM in order for them to be closer to DM images.

Future research should aim to compare the performance of DM and SM with other imaging modalities, as this would provide a more comprehensive understanding of their relative effectiveness in breast cancer diagnosis. Overall, our results demonstrate that 2D DM outperforms 2D SM in terms of cancer detection and diagnostic accuracy. The findings of our study support the growing evidence that DM is superior to SM in terms of cancer visibility and diagnostic accuracy. These results have important implications for clinical practice, as accurate and timely detection of breast cancer is crucial for successful treatment outcomes.

In conclusion, although SM demonstrated comparable performance in certain aspects, this study’s findings indicate that DM remains superior in diagnostic accuracy and cancer visibility. Additionally, there was insufficient agreement in size assessment, underscoring the need for improved lesion measurement techniques. While SM proves more effective in detecting calcifications, DM continues to outperform it in overall diagnostic accuracy and lesion visibility. Therefore, SM cannot be considered a full replacement for DM in breast cancer screening. Enhancing SM’s image quality through technological advancements is recommended, and further large-scale studies are necessary to corroborate these findings.

## Figures and Tables

**Table 1:** Sample characteristics (*n* = 375).

Characteristic	*n* (%)
Age	
Median (IQR)	52 (12)
Symptomatic	
Yes	122 (32.5%)
No	181 (48.3%)
Unknown	72 (19.2%)
Family history of breast cancer	
Yes	72 (19.2%)
No	230 (61.3%)
Unknown	73 (19.5%)
Number of children	
None	44 (11.7%)
1–3	76 (20.3%)
4–6	117 (31.2%)
7 or more	64 (17.1%)
Unknown	74 (19.7%)

**Table 2:** Diagnostic accuracy measures for DM and SM for 57 cancer cases and 290 normal women.

Measure	DM		SM	
	Value	95% CI	Value	95% CI
Sensitivity	82.46%	70.09% to 91.25%	78.95%	66.11% to 88.62%
Specificity	77.59%	72.34% to 82.25%	77.59%	72.34% to 82.25%
Positive Likelihood Ratio	3.68	2.88 to 4.70	3.52	2.74 to 4.53
Negative Likelihood Ratio	0.23	0.13 to 0.40	0.27	0.16 to 0.45
Positive Predictive Value	41.96%	36.13% to 48.03%	40.91%	34.97% to 47.13%
Negative Predictive Value	95.74%	92.74% to 97.54%	94.94%	91.87% to 96.89%
Accuracy	78.39%	73.68% to 82.60%	77.81%	73.07% to 82.07%

**Table 3:** Comparison in the number of women in each category of cancer conspicuity, density, and cancer size.

	DM	SM	*p* value
Cancer Conspicuity (visibility) *n* = 57			
Not visible	4	2	<0.05
Barely visible	2	3	
Visible, not well seen	6	16	
Clearly visible	45	36	
Breast density *n* = 375			
Non-dense (A and B)	227	202	<0.05
Dense (C and D)	148	173	
Cancer size *n* = 57			
cm	22	8	<0.05
>1–2 cm	10	13	
>2 cm	3	16	
Not measured	22	20	
Presence of cancerous microcalcification *n* = 41			
Yes	21	31	<0.05
No	20	10	

**Table 4:** Agreement between DM and SM using weighted kappa.

	*n*	Weighted Kappa (k)	95% CI
Cancer Conspicuity	57	0.288	0.065–0.511
Breast density	375	0.591	0.509–0.673
Cancer size	57	0.254	0.103–0.406
Presence of cancerous microcalcification	41	0.409	0.199–0.618

## Data Availability

If required, data can be obtained from the corresponding author.
